# A Proposed Cross-Feeding Model of Phenanthrene Degradation by Constructed Halophilic Consortium NOMA

**DOI:** 10.3390/microorganisms14081833

**Published:** 2026-08-19

**Authors:** Haoze Lu, Yilong Wen, Lin Wang, Ziheng Liang, Chongyang Wang

**Affiliations:** Miami College, Henan University, Kaifeng 475000, China; luhaoze@henu.edu.cn (H.L.); 2310380114@henu.edu.cn (Y.W.); wanglin@henu.edu.cn (L.W.); 2310380086@henu.edu.cn (Z.L.)

**Keywords:** constructed consortium, phenanthrene, genome, cross-feeding, 1-hydroxy-2-naphthoic acid

## Abstract

The accumulation of polycyclic aromatic hydrocarbons (PAHs) in saline environments poses a threat to ecological security; however, the elimination of PAHs from saline environments is difficult. In this study, a constructed microbial consortium, designated NOMA, was constructed from *Novosphingobium* sp. J3 and *Martelella* sp. E4 via a “bottom-up” method. Consortium NOMA degraded phenanthrene within 7 days at 5% salinity and retained phenanthrene-degradation activity across the tested salinity, pH, and Cd^2+^ gradients (pH values of 5–10, salinities of 1–20%, and Cd^2+^ concentrations of 0–50 mg/L). PAH-degrading genes in both strains were annotated based on the genomic information. Based on the genomic information and intermediate detection, a cross-feeding model of the phenanthrene degradation process by consortium NOMA was proposed. Strain J3 was responsible for the upstream degradation of phenanthrene, and strain E4 promoted multiple downstream degradation pathways to increase the rate of intermediate transfer. Genome-scale metabolic model (GSMM) predictions suggested a potential interspecies cross-feeding mechanism: J3 supplies upstream aromatic intermediates, whereas E4 provides complementary nutritional and cofactor-related support to J3. This study deepens the understanding of the cross-feeding pattern of PAHs in saline environments and provides a biological resource for the bioremediation of PAH under saline and cadmium stress conditions.

## 1. Introduction

Polycyclic aromatic hydrocarbons (PAHs) are persistent organic pollutants characterized by two or more fused benzene ring structures in linear, angular, or clustered arrangements [1,2,3]. Phenanthrene, a representative low-molecular-weight PAH, poses serious threats to both the environment and human health, owing to its pronounced teratogenic, carcinogenic, and mutagenic properties [4,5]. Driven primarily by anthropogenic activities, including marine oil spills, urban runoff, and petroleum-related industrial emissions, PAHs are being increasingly accumulated in saline and hypersaline environments [6]. Conventional physical and chemical remediation methods, such as solvent extraction or incineration, face severe constraints, such as high costs, incomplete contaminant removal, and secondary pollution risks [7]. Biological treatment is an economically effective method for treating persistent organic pollution. However, biological treatment faces challenges in saline environments, such as increased osmotic pressure, decreased dissolved oxygen concentration, and reduced PAH solubility. This stress disrupts normal metabolic functions and reduces the degradation rates in traditional microbial species, restricting their remediation efficacy [8]. Owing to their good salinity tolerance and wide environmental adaptability, moderate halophilic bacteria are considered high-quality microbial resources for the remediation of PAH pollution in saline environments [9,10].

Although researchers have reported several moderately halophilic degraders of PAHs and demonstrated their salinity tolerance, pure cultures have relatively low degradation rates and efficiency in PAH-degrading processes [11]. Microbial cooperation, driven by synergistic interactions among various microorganisms, can increase degradation efficiency and represents a potent, cost-effective remediation strategy [12]. Based on the theory of microbial cooperation, many artificially constructed consortia are assembled via “top-down” or “bottom-up” methods [13]. Constructed consortia present three primary advantages in terms of phenanthrene elimination. First, functional compartmentalization across multiple species minimizes metabolic crosstalk, facilitating the simultaneous execution of complex biochemical tasks [14]. Second, this division of labor mitigates individual metabolic burdens and broadens the available genomic modification space, dramatically amplifying the overall catabolic capacity [15]. Third, establishing dynamic intercellular equilibrium grants the consortium superior resilience against environmental fluctuations and high functional redundancy [16]. Therefore, leveraging constructed biology to govern metabolite exchange allows for the rational design of defined consortia, yielding optimized metabolic fluxes and robust collective performance [17]. Yang et al. engineered a tristrain consortium that completely degraded phenanthrene, with two specialized strains achieving 11.5% and 25.18% faster substrate turnover. Through synergy, the consortium also depleted a key inhibitory intermediate 12 h faster, demonstrating a highly efficient and rapid degradation system [18]. Sun et al. demonstrated that a constructed coculture of *Rhodococcus* sp. WB9 and *Mycobacterium* sp. WY10 completely degraded 100 mg/L phenanthrene within only 36 h. This remarkably rapid degradation and environmental tolerance were driven by metabolic cross-feeding, preventing toxic metabolite accumulation and synergistically enhancing the efficiency of the consortium’s bioremediation [19]. Chen et al. constructed a bacterial consortium capable of simultaneously and rapidly degrading coexisting PAHs and phthalates with remarkable efficiency. This environmental tolerance in complex matrices, such as copolluted soils and sludges, was achieved by enriching specific functional taxa and altering extracellular polymeric substances to synergistically increase cometabolism [20].

Despite the increasing application of constructed microbial consortia for PAH degradation, the strain-resolved metabolic basis underlying their enhanced performance in saline environments remains insufficiently understood. In particular, how different members coordinate upstream PAH transformation, downstream aromatic metabolism, and potential metabolite exchange has not been fully elucidated. A clearer understanding of these interactions is important for the rational design of defined microbial consortia with improved degradation capacity and environmental adaptability. Therefore, in the present study, a defined halophilic consortium composed of *Novosphingobium* sp. J3 and *Martelella* sp. E4 was constructed to investigate phenanthrene degradation under saline conditions. The degradation performance and environmental responses of the consortium were characterized, while whole genome analysis and GC–MS metabolite profiling were used to investigate the potential cross-feeding degradation pathways. Genome-scale metabolic modeling was further employed to evaluate the potential metabolic complementarity between the two strains. This study aimed to provide a mechanistic framework for understanding cooperative PAH degradation in defined microbial consortia and to support the development of microbial resources for PAH-contaminated saline environments.

## 2. Materials and Methods

### 2.1. Isolation and Cultivation of Degrading Bacteria

Two bacterial strains, designated J3 and E4, were isolated from a previously established PAH-degrading consortium 5H [9]. The sea salt-defined medium (SSDM) was composed of the following basal components at a salinity of 5%: KCl (2.1 g/L), (NH_4_)_2_SO_4_ (2.0 g/L), NaHCO_3_ (0.1 g/L), CaCl_2_ (0.75 g/L), NaCl (39.72 g/L), MgCl_2_·6H_2_O (3.45 g/L), and MgSO_4_·7H_2_O (5.22 g/L). This medium was supplemented with 1 mL/L each of a trace element solution, vitamin solution I, and vitamin solution II, and the initial pH was adjusted to 7.0−7.3. For strain isolation, the consortium suspension was serially diluted and spread onto two types of solid SSDM agar plates: SSDM-P, containing 100 mg/L phenanthrene as the sole carbon source, and SSDM-Y, containing 100 mg/L yeast extract as the sole carbon source, respectively. After incubation at 30 °C for 5 to 7 days, strain J3 was purified from colonies producing clear zones on SSDM-P, and strain E4 was purified from morphologically distinct colonies on SSDM-Y, both by repeated streaking. After isolation, strain J3 was further cultured using 200 mL SSDM at 5% salinity, using 100 mg/L phenanthrene as the sole carbon source. Strain E4 was cultured in 200 mL SSDM at 5% salinity using 100 mg/L yeast extract as the sole carbon source. Both strains were cultured in darkness, at 30 °C, 120 rpm. Cells were harvested at the late-exponential growth phase, washed with fresh 5% SSDM, for DNA extraction or subculturing.

### 2.2. Genomic DNA Extraction, Sequencing, and Functional Annotation

Genomic DNA from strain J3 and strain E4 were extracted from the cell suspensions obtained after a 4-day incubation period utilizing the Fast DNA Spin Kit for Soil (MP Biomedicals Co., Ltd., Santa Ana, CA, USA) following the manufacturer’s protocols. The draft genome sequences for both strains were acquired via whole-genome shotgun sequencing on the Illumina platform (Illumina, San Diego, CA, USA). Sequencing data were subsequently processed through the Galaxy Bioinformatics Analysis Platform (https://usegalaxy.org/ and https://usegalaxy.cn/, accessed on 15 august 2026). After sequencing, the raw reads were assembled and optimized for k-mer values using Velvet (version 1.2.10). Predictions of coding genes, rRNA, tRNA, and other ncRNAs for both strains were obtained with the aid of Prokka (version 1.14.6). The functions of the predicted protein-coding genes were annotated by aligning the sequences against multiple established databases, including the Kyoto Encyclopedia of Genes and Genomes (KEGG, https://www.kegg.jp/kegg/, 15 August 2026), Gene Ontology (GO), Clusters of Orthologous Groups (COG), and Carbohydrate-Active Enzymes (CAZy) databases. The full-length 16S rRNA gene sequences of strains J3 and E4 were retrieved from their assembled genome sequences and compared with reference sequences in the NCBI nucleotide database using BLASTn (BLAST v2.3.0, https://blast.ncbi.nlm.nih.gov/Blast.cgi; accessed on 15 August 2026). The draft genome assemblies of *Novosphingobium* sp. J3 and *Martelella* sp. E4 were deposited in GenBank under the accession numbers JCASKL000000000 and JCASKK000000000, respectively, under BioProject PRJNA1498824.

### 2.3. Assembly of the Constructed Microbial Consortium NOMA

Strain J3 and strain E4 were individually scaled up in SSDM supplemented with 100 mg/L phenanthrene and 100 mg/L yeast extract as previously described. Cells were harvested by centrifugation upon reaching the late exponential growth phase. The initial OD600 of strain J3 was 0.264 and the initial OD600 of strain E4 was 0.251. Cell suspensions of the two strains were collected and mixed according to their initial OD600 to obtain equal cell abundances for both strains in the mixture. Subsequently, the mixture was centrifuged and washed twice with fresh 5%-SSDM to obtain the constructed consortium NOMA. Biodegradation assays utilizing this consortium were subsequently initiated in 200 mL of SSDM (5% salinity) containing 100 mg/L phenanthrene as the sole carbon and energy source. The initial OD600 of both degradation systems, namely strain J3 pure culture and consortium NOMA, were adjusted to 0.0150. These culture systems were maintained in the dark at 30 °C with continuous agitation at 120 rpm.

### 2.4. Measurement of Phenanthrene Biodegradation Kinetics

Assays to determine phenanthrene degradation were conducted in 200 mL of 5% salinity SSDM with an initial phenanthrene concentration of 100 mg/L. The culture flasks were incubated on a rotary shaker (30 °C, 120 rpm). To monitor the degradation kinetics, 2 mL culture aliquots were collected twice daily and subjected to extraction with 5 mL of dichloromethane. These biphasic mixtures underwent vigorous rotary shaking for 30 min before filtration. Residual phenanthrene concentrations were quantified using a high-performance liquid chromatography system (HPLC; Shimadzu, Tokyo, Japan) coupled to an XB-C18 reversed-phase column (5 μm, 4.6 × 200 mm). An isocratic mobile phase of methanol and ultrapure water (90:10, *v*/*v*) was delivered at 1.0 mL/min. The separation was performed at a column temperature of 40 °C, with UV detection at 254 nm and a total run time of 20 min [9]. Phenanthrene standards were prepared in dichloromethane and serially diluted for retention-time identification and calibration-curve construction [12]. An abiotic control, comprising uninoculated medium with an identical initial phenanthrene concentration, was included to precisely account for nonbiological volatilization or loss. All experiments were performed in triplicate. Concurrently, the optical density at 600 nm (OD600) was recorded at predetermined time intervals to determine cellular growth. Naphthalene biodegradation assays were conducted in parallel under the same conditions, except that phenanthrene was replaced with naphthalene at an initial concentration of 100 mg/L. Residual naphthalene was extracted and quantified using the same procedures. The assays included an abiotic control and were performed in triplicate.

Meanwhile, 50 mg/L 1-hydroxy-2-naphthoic acid, salicylic acid, gentisate, and protocatechuic acid were utilized as the sole carbon source for the cultivation of strain J3 and strain E4, respectively, to determine the utilization ability of intermediates by these two strains.

### 2.5. Phenanthrene Degradation Under Varying Salinities, pH Conditions and Cadmium Stress Levels

To delineate the optimal biodegradation conditions for consortium NOMA, its phenanthrene-degrading capacity was evaluated across a defined gradient of salinities (1%, 3%, 5%, 10%, 15% and 20%) and pH levels (5, 6, 7, 8, 9 and 10). Target salinities were achieved by proportionally adjusting the total salt content using NaCl, MgCl_2_·6H_2_O, and MgSO_4_·7H_2_O. The initial pH of the culture medium was adjusted utilizing 0.5 mol/L NaOH and 1:5 (*v*/*v*) HCl. Three independently prepared culture flasks were used for each treatment and were considered biological replicates, and the corresponding degradation rates were systematically determined at specific time intervals. Degradation assays evaluating heavy metal stress were executed following the parameters described above. The bacterial consortium NOMA was cultivated in 200 mL of SSDM (5% salinity) with 100 mg/L phenanthrene as the sole carbon source. Cadmium was selected as a representative heavy metal stressor because cadmium and PAHs may coexist in coastal environments. Previous studies have also examined the combined effects of cadmium and phenanthrene in coastal water microcosms [21]. To simulate co-contamination stress, a stock solution of cadmium nitrate tetrahydrate (Cd(NO_3_)_2_·4H_2_O) was introduced to establish a precise gradient of Cd^2+^ concentrations: 0, 0.5, 10, 25, and 50 mg/L. Residual phenanthrene concentrations were quantified via HPLC following previously described methods. Concurrently, the physiological tolerance of the bacterial consortium under cadmium stress was measured by tracking the optical density (OD600).

### 2.6. GC–MS Detection of Intermediate Metabolites

Intermediate metabolites were detected during the phenanthrene degradation process by pooling 50 mL of cell suspensions collected during both the early (day 3) and late (day 7) degradation stages. This combined mixture underwent initial extraction with 100 mL of chromatographically pure dichloromethane in a separating funnel. After 20 min of vigorous shaking, the lower organic layer was collected. The remaining aqueous phase was acidified to a pH of 1–2 using HCl (1:5, *v*/*v*) and extracted with an additional 50 mL of dichloromethane. Finally, the aqueous phase was basified to a pH of 12–13 utilizing 2 M NaOH and subjected to a third extraction with 50 mL of dichloromethane. The combined organic extracts (approximately 200 mL) were filtered through anhydrous sodium sulfate for complete dehydration. The resulting filtrate was concentrated to approximately 5 mL using a vacuum rotary evaporator, transferred into a dry digestion tube, and completely evaporated under a gentle stream of high-purity nitrogen. Derivatization was accomplished by adding 0.1 mL of chromatographic-grade methanol, 0.1 mL of chromatographic-grade acetonitrile, and 0.1 mL of N,O-bis(trimethylsilyl)trifluoroacetamide (BSTFA) containing 1% trimethylchlorosilane (TMCS) (99:1, *v*/*v*), following previously reported procedures for GC–MS analysis of phenanthrene degradation intermediates [9,12]. The sealed tubes were incubated at 60 °C for 40 min. Upon cooling to room temperature, the derivatized samples were reconstituted in 0.7 mL of chromatographically pure dichloromethane, filtered through a 0.22 μm nylon syringe filter into 1.5 mL autosampler vials, and subsequently analyzed by gas chromatography–mass spectrometry (GC–MS). Only silylated extracts were analyzed. GC–MS analysis was performed using an Agilent 7890A gas chromatograph coupled to an inert mass selective detector with a Triple-Axis Detector (Agilent, Santa Clara, CA, USA). Separation was achieved on an HP-5 capillary column (30 m × 0.25 mm internal diameter × 0.25 μm film thickness), with helium supplied as the carrier gas at 1.0 mL/min. The injector was maintained at 250 °C. The oven was programmed from an initial temperature of 80 °C, held for 1 min, to 280 °C at a ramp rate of 5 °C/min, followed by a 10 min hold at 280 °C. Mass spectra were recorded under electron-impact ionization at 70 eV over an m/z range of 50–550. The ion source and quadrupole temperatures were set at 230 and 150 °C, respectively. Authentic standards, including 1-hydroxy-2-naphthoic acid, phthalic acid, salicylic acid, and catechol, were subjected to the same derivatization and GC–MS conditions for compound identification.

### 2.7. Computational Reconstruction of Genome-Scale Metabolic Models (GSMMs)

The Prokka-predicted protein sequences of *Novosphingobium* sp. J3 and *Martelella* sp. E4 were used to reconstruct draft genome-scale metabolic models (GSMMs) using CarveMe (version 1.5.2) through the iNAP2 platform. The biomass reactions generated by CarveMe were retained as the biomass objectives. The initial J3 model contained 1814 reactions and 1341 compounds, whereas the initial E4 model contained 2224 reactions and 1647 compounds. No additional gap filling was performed during draft reconstruction.

Because the automatically reconstructed models incompletely represented the specialized aromatic hydrocarbon degradation pathways identified by genomic annotation, the draft models were manually curated using the KBase Edit Metabolic Model workflow. Missing reactions were incorporated based on strain-specific genomic annotations and established reactions and compounds in the ModelSEED and KEGG databases. The final curated SBML models were subsequently analyzed using the standalone implementation of PhyloMInt to evaluate pairwise metabolic complementarity and potentially transferable metabolites (PTMs). PTMs were further classified according to their cellular compartments, and extracellularly represented metabolites were used for the main interpretation of potential interstrain metabolic exchange.

### 2.8. Statistical Analysis

All biodegradation experiments were performed using three independently prepared culture flasks for each treatment and were considered biological replicates (*n* = 3). Quantitative data are presented as the mean ± standard deviation (SD) or the raw parallel data directly, and error bars in the corresponding figures represent SD.

## 3. Results and Discussion

### 3.1. Identification of Consortium NOMA

**Figure 1:**
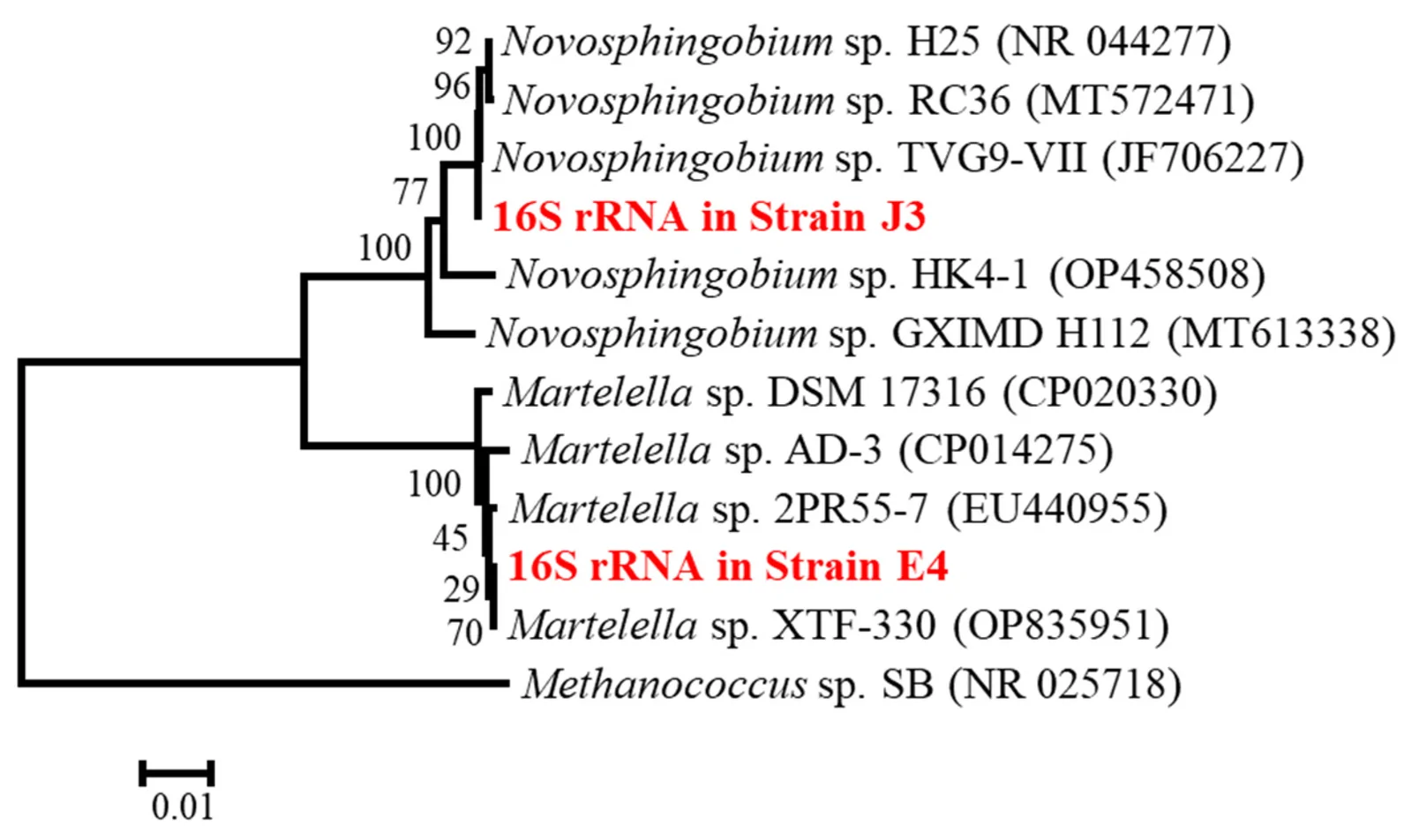
Phylogenetic analysis of strains J3 and E4 based on 16S rRNA gene sequences.

Full-length 16S rRNA gene sequences were retrieved from the assembled genomes of strains J3 and E4 and subjected to homology analysis against the NCBI nucleotide database. As shown in Figure 1, phylogenetic tree construction confirmed the taxonomic status of the 2 isolates as *Novosphingobium* and *Martelella*. Therefore, these 2 strains were named *Novosphingobium* sp. J3 and *Martelella* sp. E4, respectively. Both genera are frequently detected in PAH-polluted marine environments and are well documented for their metabolic potential. For instance, *Novosphingobium* sp. US6-1 rapidly degraded 86.62% of phenanthrene within 24 h [22]. Similarly, *Martelella* sp. AD-3 was capable of complete phenanthrene degradation under optimal saline conditions (3% salinity, pH 9.0) [23].

Strain J3 was shown to be able to degrade phenanthrene alone with a relatively slow degradation rate, as it was able to remove 100 mg/L phenanthrene in 10 days. Strain E4 showed no ability to degrade phenanthrene alone. Therefore, to increase the degradation capacity of strain J3, the constructed consortium NOMA was constructed.

### 3.2. Degradation Ability of the Artificial Consortium NOMA

**Figure 2:**
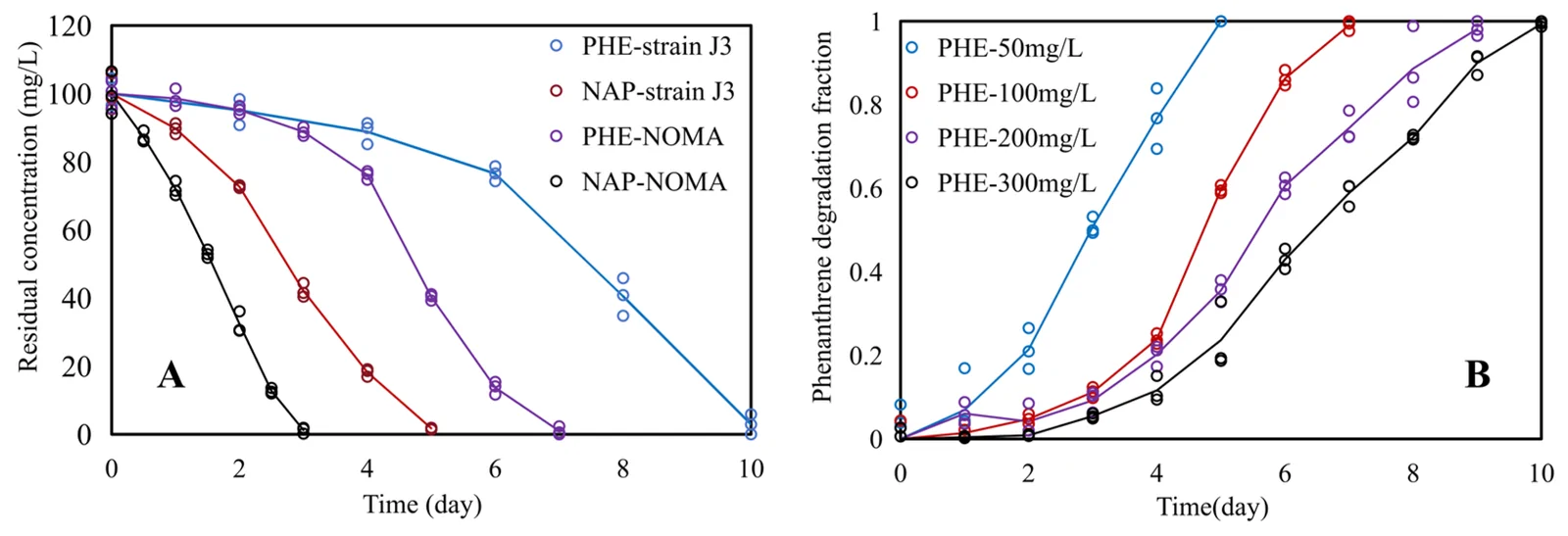
Degradation profiles of phenanthrene and naphthalene. (**A**) Residual concentrations of phenanthrene and naphthalene during degradation by strain J3 and consortium NOMA. (**B**) Phenanthrene degradation fractions of consortium NOMA at different initial phenanthrene concentrations under 5% salinity.

At 5% salinity, strain J3 was able to completely degrade 100 mg/L phenanthrene in 10 days and 100 mg/L naphthalene in 5 days. With the addition of strain E4, the degradation rate of naphthalene and phenanthrene was increased (Figure 2A). The constructed consortium NOMA achieved complete removal of phenanthrene in 7 days and 100 mg/L naphthalene in 3 days. Kinetic analysis showed that the apparent zero-order model provided the best fit for phenanthrene degradation by both strain J3 and consortium NOMA, with rate constants of 9.45 and 15.42 mg·L^−1^·d^−1^ and half-lives of 5.29 and 3.24 d, respectively. The apparent degradation rate constant of NOMA was therefore 1.63-fold that of J3 alone, corresponding to a 63.1% kinetic enhancement. The degradation rate of phenanthrene by consortium NOMA was detected at 5% salinity, with initial phenanthrene concentrations ranging from 50–300 mg/L (Figure 2B). The consortium successfully achieved 100% degradation across all tested thresholds, underscoring its exceptional bioremediation efficacy. At low concentrations (50 and 100 mg/L), the phenanthrene degradation rate of the consortium was rapid and did not have a perceptible lag phase, and the phenanthrene was completely removed within 5 and 7 days, respectively. As the pollutant load increased to 200 mg/L and 300 mg/L, complete degradation was achieved in 9 and 10 days, respectively. A pronounced lag phase was observed during the initial 48 h at 300 mg/L. Following this acclimation, the subsequent exponential decay confirmed that the synergistic interactions within NOMA effectively increased the rate of phenanthrene removal.

**Figure 3:**
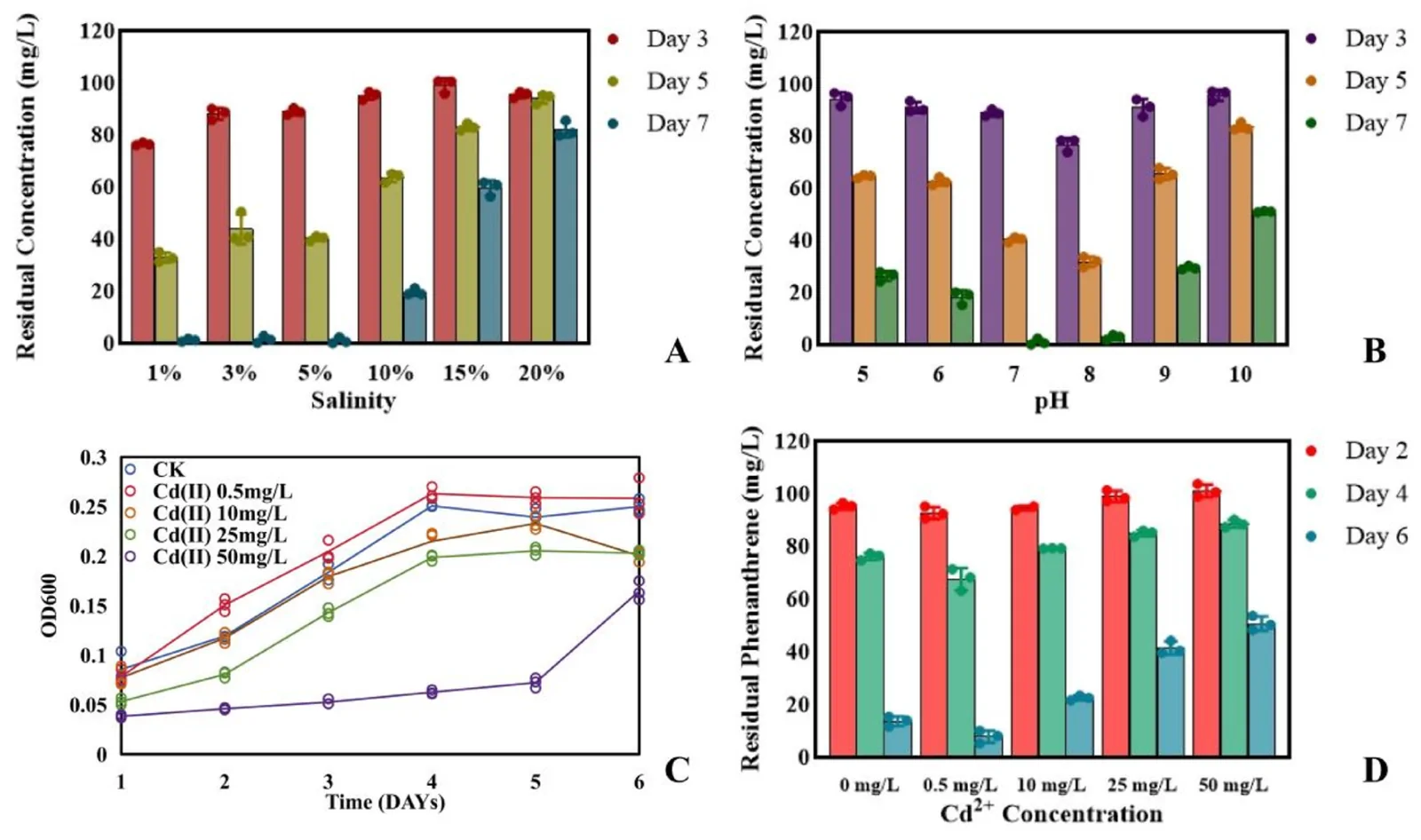
Effects of environmental conditions on phenanthrene degradation by consortium NOMA. (**A**) Residual phenanthrene concentrations under different salinities (1%, 3%, 5%, 10%, 15%, and 20%). (**B**) Residual phenanthrene concentrations under different initial pH values (5–10). (**C**) Growth of consortium NOMA under different Cd^2+^ concentrations. (**D**) Residual phenanthrene concentrations under different Cd^2+^ concentrations. Data are presented as mean ± SD of three independent biological replicates (*n* = 3).

Environmental salinity typically induces severe osmotic pressure that disrupts normal metabolic functions and reduces degradation kinetics. However, consortium NOMA demonstrated fairly strong resilience. Over a 7-day incubation period, the consortium exhibited efficient degradation across a low-to-moderate salinity range (1–5%), and the residual phenanthrene concentration was nearly zero (Figure 3A). Although a 10% salinity level imparted a slight inhibitory effect, substantial catabolic activity was maintained (the residual concentration was approximately 20 mg/L). Only under hypersaline conditions was the degradative activity severely suppressed. Consistent with this trend, the apparent rate constant decreased from 14.51 mg·L^−1^·d^−1^ at 5% salinity to 2.33 mg·L^−1^·d^−1^ at 20% salinity, while the corresponding half-life increased from 3.45 to 21.43 d (Table S6). The consortium also demonstrated broad pH plasticity, successfully removing phenanthrene at pH values between 5.0 and 10.0 (Figure 3B). Optimal catabolism occurred under neutral to weakly alkaline conditions (pH 7.0–8.0), whereas highly acidic (pH 5.0) and highly alkaline (pH 10.0) extremes partially hindered the enzymatic cascade. These results indicate that NOMA tolerates a range of salinity and pH conditions under defined laboratory conditions, while its degradation performance declines under more extreme conditions.

The growth dynamics of the constructed consortium NOMA cultivated under varying cadmium (Cd^2+^) concentrations, utilizing phenanthrene as the sole carbon source, demonstrated a highly resilient, concentration-dependent physiological response. As shown in Figure 3C, the control (0 mg/L Cd^2+^) exhibited typical growth kinetics, reaching the stationary phase by day 4 with a maximum optical density (OD600) of approximately 0.25. Notably, compared with the control treatment, exposure to a low concentration of Cd^2+^ (0.5 mg/L) resulted in a slight increase in final biomass accumulation, consistent with previous reports that very low cadmium concentrations stimulated DNA synthesis and cell growth [24,25], resulting in slightly greater final biomass accumulation. At moderate Cd^2+^ concentrations (10 and 25 mg/L), dose-dependent inhibition occurred, characterized by a suppressed initial growth rate. Nevertheless, the consortium maintained robust cellular viability, successfully entering the exponential phase and recovering to near-control biomass levels (OD600 > 0.20) by days 4 and 5. The recovery of microbial growth under Cd^2+^ exposure indicates that consortium NOMA retained physiological activity under the tested metal-stress conditions, which are likely mediated by heavy metal efflux pumps (Czc systems and P-type ATPases), which actively prevent severe intracellular damage. Under extreme Cd^2+^ stress (50 mg/L), consortium NOMA experienced a prolonged lag phase, with cellular growth remaining suppressed for the first 5 days. Strikingly, this was followed by sharp exponential proliferation between days 5 and 6. Rather than indicating irreversible toxicity, this delayed yet full recovery suggests that the extended lag phase provided a critical temporal window for the consortium to upregulate its intrinsic heavy metal resistance mechanisms and resumed the efficient, synergistic biodegradation of phenanthrene.

Under Cd^2+^ stress at varying concentrations, the phenanthrene degradation rate of consortium NOMA was consistent with its growth pattern (Figure 3D). The addition of a low concentration of Cd^2+^ increased the phenanthrene degradation rate by consortium NOMA. When the concentration of Cd^2+^ increased above 10 mg/L, the degradation rate of PAHs by the microbial consortium NOMA was inhibited. This phenomenon may be attributed to the suppression of cell growth under high Cd^2+^ stress. Consistent with these observations, the apparent rate constant reached its maximum of 15.08 mg·L^−1^·d^−1^ at 0.5 mg/L Cd^2+^ and progressively decreased to 8.01 mg·L^−1^·d^−1^ at 50 mg/L Cd^2+^. Detailed kinetic parameters and R^2^ values are provided in Table S6.

### 3.3. Genomic Sequencing and Analysis of NOMA Strains

**Figure 4:**
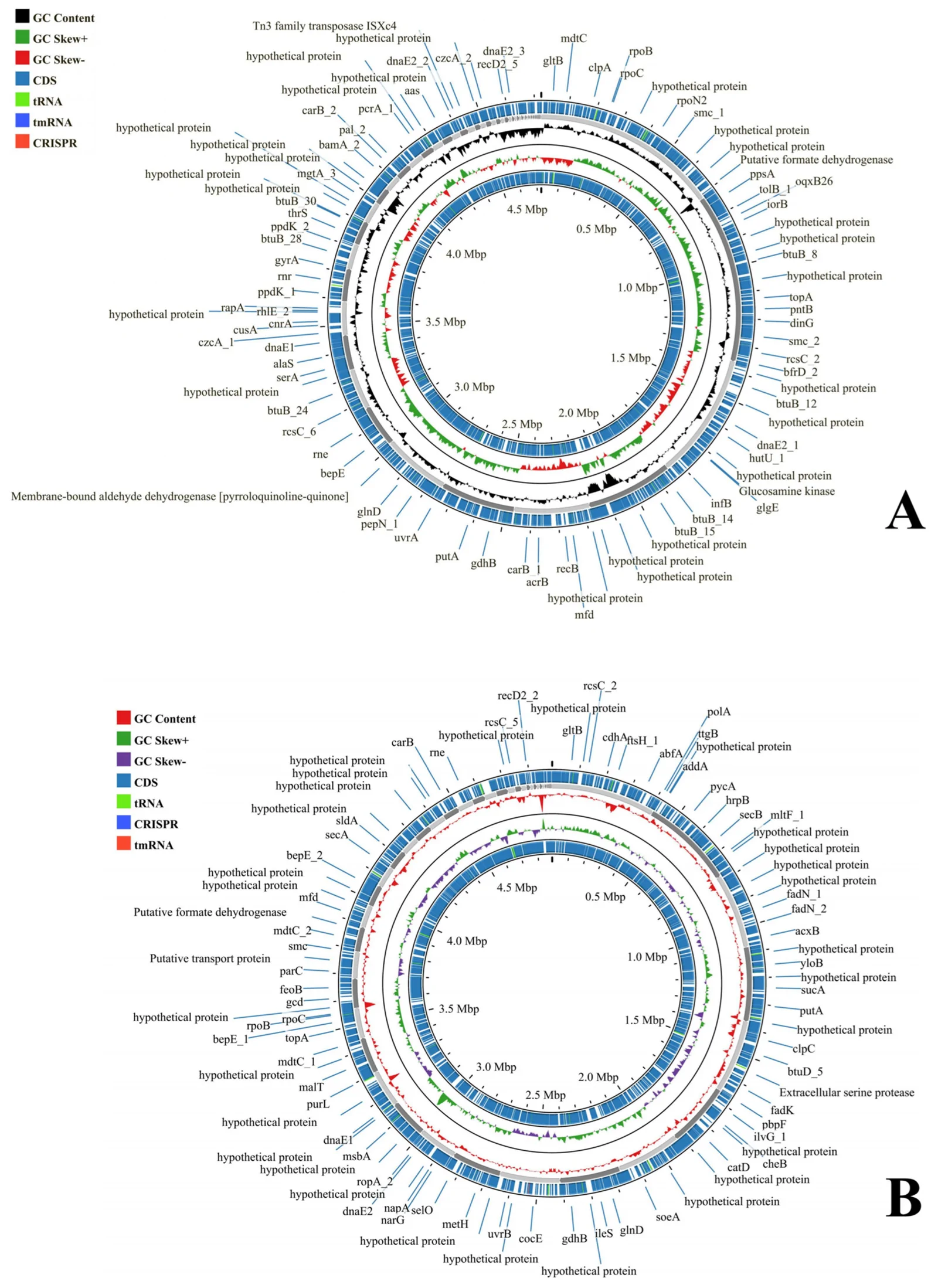
Genomic map of (**A**) *Novosphingobium* sp. J3 and (**B**) *Martelella* sp. E4.

Circular genome maps provide a robust framework for visualizing the structural architecture and functional potential of genomic data. Whole-genome sequencing and de novo assembly yielded draft genome assemblies of 4,724,468 bp for *Novosphingobium* sp. J3 and 4,886,235 bp for *Martelella* sp. E4. The J3 and E4 assemblies comprised 59 and 53 contigs, with contig N50 values of 289,018 bp and 237,366 bp, respectively. The mean sequencing depths of strains J3 and E4 were 205.58× and 261.11×, respectively, with genome coverage values of 100% and 100%. The GC contents of genomes of strain J3 and E4 were 63.06% and 62.51%, respectively. Draft assemblies were visualized as circular genome maps using Proksee (https://proksee.ca/, accessed on 26 March 2026) (Figure 4). GC skew, a measure of strand-specific nucleotide composition bias that can reflect replication-related genomic features, was calculated using the (G − C)/(G + C) algorithm. Subsequent annotation via Prokka (version 1.14.6) revealed a total of 4624 protein-coding sequences (CDSs) in the genome of strain J3 and 4562 CDSs in the genome of strain E4. Spatial distribution analysis revealed that functional genes are distributed evenly and densely across chromosomes. In contrast, there are significantly fewer noncoding elements, including tRNAs, CRISPR arrays, and tmRNAs, and these elements are sparsely distributed. Crucially, a substantial proportion of the annotated genes encode hypothetical proteins with unknown functions. These open reading frames suggest vast, untapped metabolic potential; however, their specific biological roles and contributions to the synergistic degradation network require further experimental verification.

### 3.4. PAH-Degrading Genes and Heavy Metal Resistance Genes in Artificial Consortium NOMA

**Figure 5:**
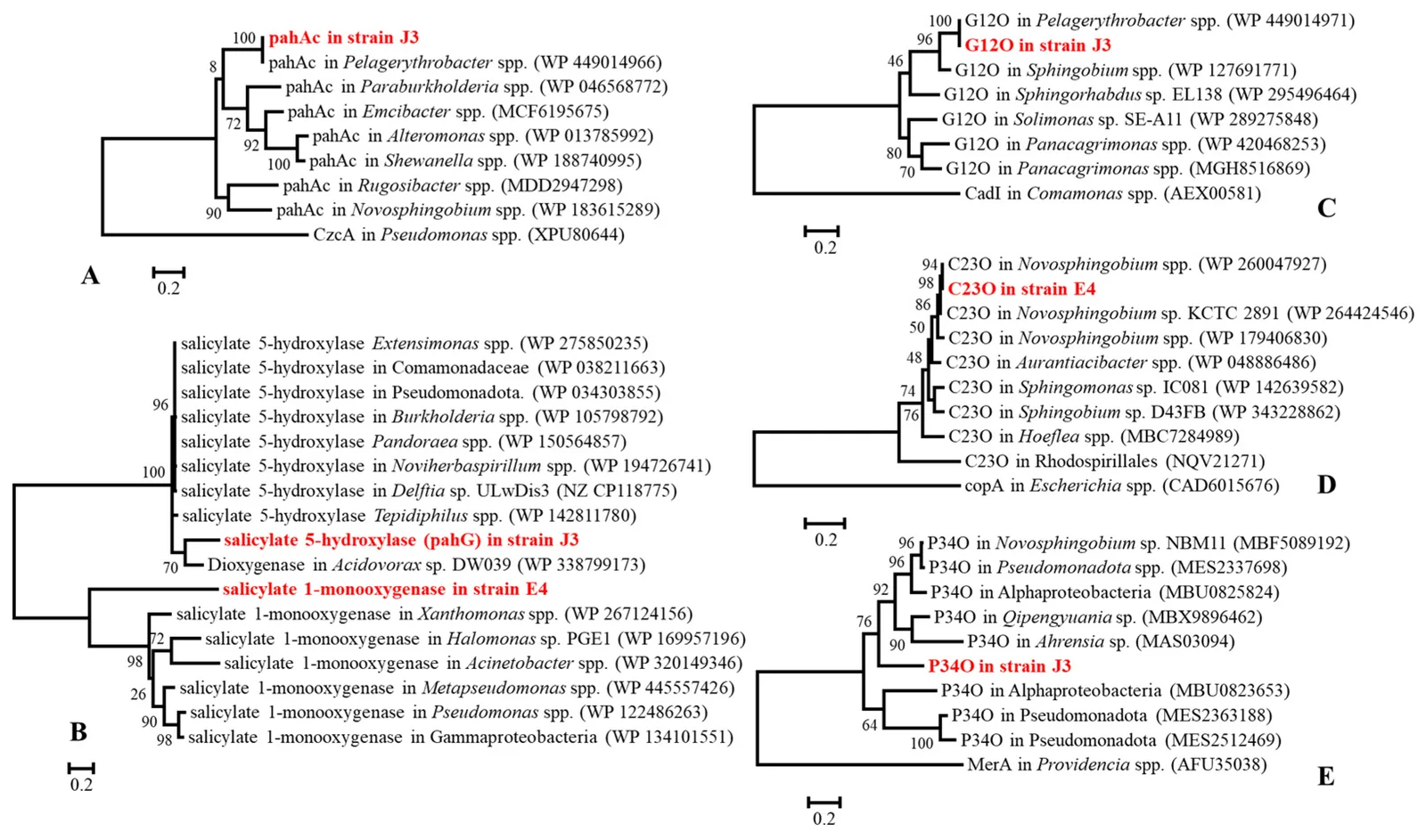
PAH-degrading related enzymes contained in NOMA (**A**) *pahAc* in strain J3, (**B**) *pahG* in strain J3 and E4, (**C**) *G12O* in strain J3, (**D**) *C23O* in strain E4, (**E**) *P34O* in strain J3.

During the aerobic biodegradation of PAHs, aromatic ring-hydroxylating dioxygenases (RHDs) initiate the activation of aromatic rings by incorporating oxygen atoms into one or more carbon atoms of the ring structure. This initial step is rate-limiting in aerobic biodegradation, fundamentally determining the potential and reaction kinetics of subsequent microbial metabolism [26]. In this study, the *pahAc* gene, which encodes the alpha subunit of RHDs, was identified in the genome of *Novosphingobium* sp. J3 by comprehensive genome annotation and analysis. The amino acid sequence of RHD in *Novosphingobium* sp. J3 was 100% similar to that of RHD in *Pelagerythrobacter* spp. (WP_449014966) and 58.22% similar to that of RHD in *Rugesibacter* spp. (MDD2947298) (Figure 5A). *Martelella* sp. E4 harbored no RHD-encoding genes, indicating that strain E4 cannot initially transform phenanthrene. The genetic repertoire indicated that consortium NOMA possesses phenanthrene degradation capabilities, enabling aromatic ring cleavage in the upstream pathway to facilitate subsequent catabolism through strain J3.

The *pahG’* gene in the genome of strain J3, which encodes salicylate-5-hydroxylase, was identified. A phylogenetic tree of the amino acid sequence of salicylate-5-hydroxylase in strain J3 is shown in Figure 5B. Moreover, genes encoding gentisate 1,2-dioxygenase (G12O) in the genome were also annotated (Figure 5C). The presence of salicylate-5-hydroxylase and G12O indicates that strain J3 can further transfer the downstream intermediate salicylate to gentisate and cleave the last benzene ring through the G12O pathway [27]. No genes encoding RHDs such as enzymes or other PAH-encoding genes upstream of degradation were identified in the genome of strain E4. Because initial aromatic-ring activation represents a critical step in PAH biodegradation [13], the absence of the identified upstream PAH-degrading genes suggests that strain E4 lacks the genetic capacity to initiate phenanthrene degradation through this pathway.

In the genome of strain E4, the *pahG* gene encoding salicylate 1-monooxygenase (Figure 5B) and the *cat* gene encoding catechol 2,3-dioxygenase (C23O, Figure 5D) were identified. In phenanthrene degradation, salicylate 1-monooxygenase catalyzes the hydroxylation processes from 1-hydroxy-2-naphthoic acid to 1,2-dihydroxynaphthalene and from salicylic acid to catechol [28]. The presence of salicylate 1-monooxygenase and C23O indicated that strain E4 can transfer salicylate to catechol and further cleave catechol through the C23O pathway [29].

Furthermore, gene annotation indicated that strain J3 likely possesses an additional phenanthrene degradation pathway. In this route, the intermediate 1-hydroxy-2-naphthoic acid undergoes direct ring cleavage, resulting in the formation of phthalic acid via a novel pathway. Phthalic acid is then converted to protocatechuic acid, and the final aromatic ring is cleaved by protocatechuate 3,4-dioxygenase (P34O, Figure 5E). In summary, although the upstream degradation pathways of PAHs are relatively conserved, the downstream pathways exhibit considerable diversity. At the genomic level, consortium NOMA encompasses three known downstream degradation pathways. Moreover, the genomic annotations suggest that strain J3 possesses multiple downstream pathways for the metabolism of PAH-derived intermediates. This genetic redundancy and metabolic versatility explain the outstanding phenanthrene degradation performance of consortium NOMA. Other PAH-degrading genes in consortium NOMA are listed in Table S1.

**Figure 6:**
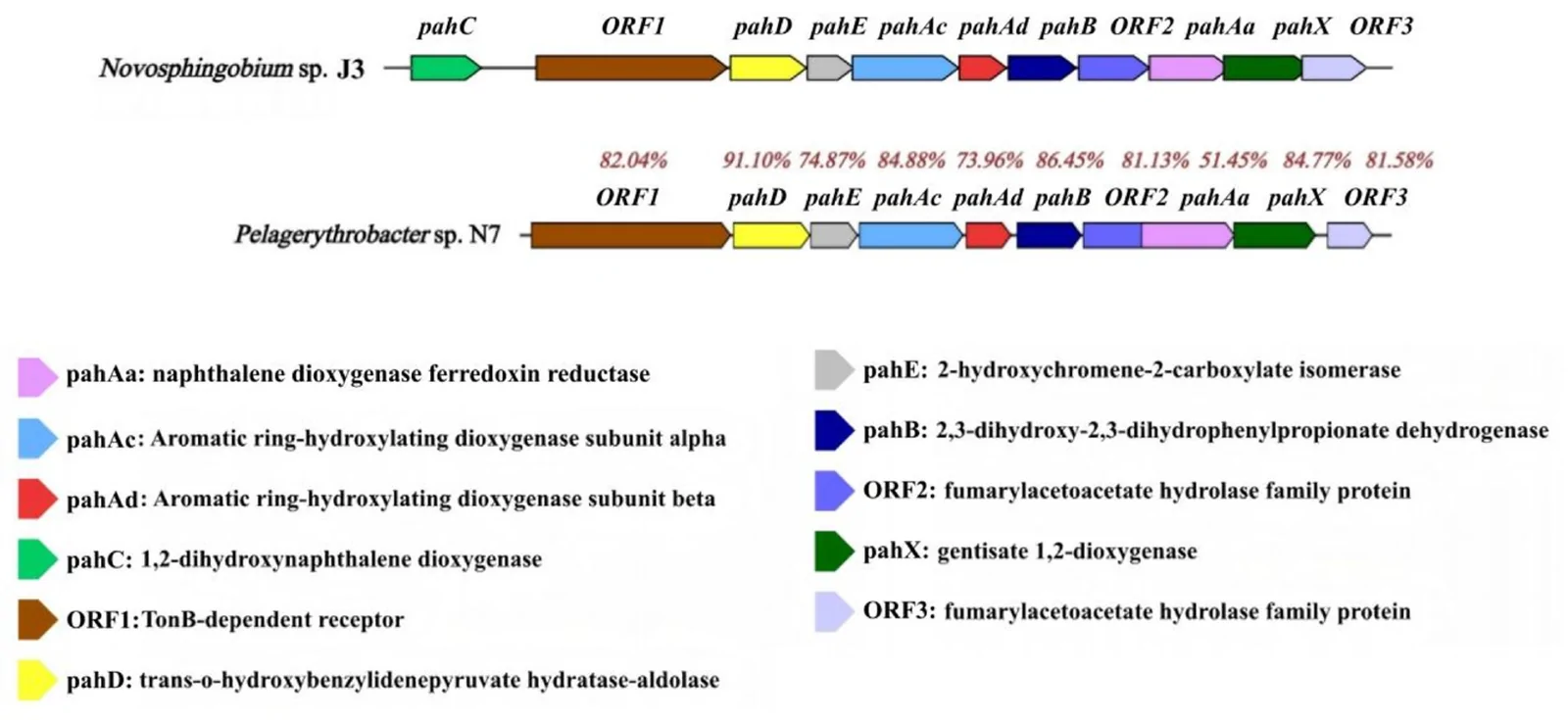
Physical map and genetic arrangement of the gene clusters in *Novosphingobium* sp. J3. Related cluster in *Pelagerythrobacter* sp. N7 was illustrated for comparison.

The upstream phenanthrene-degrading gene cluster in strain J3 was identified and is illustrated in Figure 6. This PAH-degrading gene cluster contained 11 ORFs (Table S2). The protein encoded by *pahC*, which is 301 amino acids in size, showed 62.16% similarity with the allele in Pseudomonas (WP_141123765) and was identified as a 1,2-dihydroxynaphthalene dioxygenase. The protein encoded by ORF1, which is 824 amino acids in size, showed 82.29% similarity with the allele in *Sphingobium* (WP_241242031) and was identified as a TonB-dependent receptor. The protein encoded by *pahD* is a 2-hydroxychromene-2-carboxylate isomerase with a length of 199 amino acids and exhibits 100% similarity to 2-hydroxychromene-2-carboxylate isomerase in *Pelagerythrobacter* (WP_449014965). The protein encoded by *pahAc*, which is 458 amino acids in size, also showed 100% similarity with the allele in *Pelagerythrobacter* (WP_449014966) and was identified as an aromatic ring-hydroxylating dioxygenase subunit alpha. Moreover, genes related to PAH degradation, such as *pahE* and *pahB*, exhibited a highly clustered arrangement in this cluster. The PAH-degrading gene cluster in strain J3 was highly similar to the gene cluster in *Pelagerythrobacter* sp. N7 in terms of both the gene cluster arrangement and the amino acid sequence of each ORF, suggesting possible horizontal gene transfer or a shared evolutionary origin between the two strains.

In consortium NOMA, a substantial number of heavy metal resistance genes were identified based on the alignment results from the BacMet database [30]. The protein encoded by *cadA*, which is 707 amino acids in size, was 93.06% similar to the corresponding allele in *Microvirga* (MCG6122998.1) and was identified as a cadmium-translocating P-type ATPase. The protein encoded by *czcA*, which is 1045 amino acids in size, showed 85.80% similarity with the allele in *Pseudomonadota* (MED5545921.1) and was identified as a CusA/CzcA family heavy metal efflux RND transporter. In addition, typical metal resistance genes, such as *zntA*, *copA*, and *merA*, were also annotated in NOMA. The presence of these annotated heavy-metal resistance genes indicates the potential genetic basis for the observed Cd^2+^ tolerance of consortium NOMA, as shown in Table S3.

### 3.5. Prediction of the PAH Degradation Pathway in NOMA

**Figure 7:**
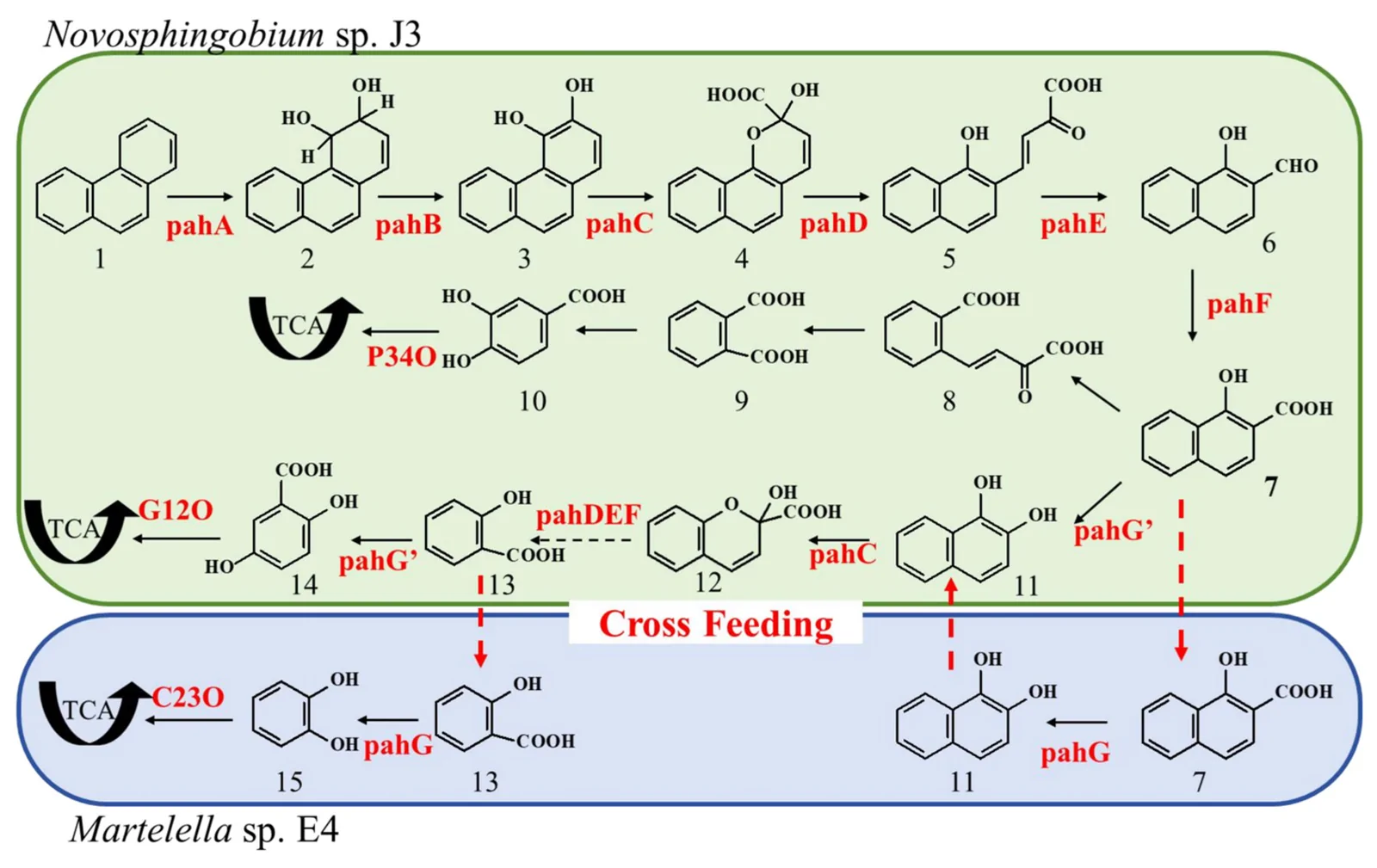
Degradation pathway of phenanthrene by constructed microbial consortium NOMA (**1**: phenanthrene, **2**: cis-3,4-dihydroxy-3,4-dihydrophenanthrene, **3**: 3,4-dihydroxyphenanthrene, **4**: 2-hydroxy-2H-benzo(h)chromene-2-carboxylic, **5**: trans-4-(1-hydroxynaph-2-yl)-2-oxobut-3-enoic acid, **6**: 1-hydroxy-2-naphthoaldehyde, **7**: 1-hydroxy-2-naphthoic acid, **8**: trans-2′-carboxybenzalpyruvic acid, **9**: o-phthalic acid, **10**: protocatechuic acid, **11**: 1,2-dihydroxynaphthalene, **12**: 2-hydroxy-2H-chromene-2-carboxylic acid, **13**: salicylic acid, **14**: gentisic acid, **15**: catechol).

The metabolic intermediates of phenanthrene degradation in NOMA were detected using GC–MS. As shown in Table S4, key phenanthrene degradation intermediates, including 1-hydroxy-2-naphthoic acid, salicylic acid, catechol, gentisate, etc., were successfully detected. Meanwhile, as shown in Table 1, strain J3 was identified as being able to utilize phenanthrene, 1-hydroxy-2-naphthoic acid, salicylic acid, gentisate and protocatechuic acid. Strain E4 was identified as being able to utilize salicylic acid and catechol. Based on the results of intermediates utilization ability, GC–MS, and gene annotations, a cross-feeding degradation pathway for consortium NOMA was proposed, as shown in Figure 7. The upstream degradation of phenanthrene by consortium NOMA was driven by the PAH-upstream degrading gene cluster contained in strain J3. The RHDs in the gene cluster catalyze a crucial double-oxygenation reaction on the aromatic ring to produce cis-3,4-dihydroxy-3,4-dihydrophenanthrene. Under the sequential enzymatic action of *pahB*, *pahC*, *pahD*, *pahE*, and *pahF*, this intermediate is subsequently converted to 1-hydroxy-2-naphthoic acid. 1-hydroxy-2-naphthoic acid was the first cross-feeding intermediate and can be further transferred to 1,2-dihydroxynaphthalene via both the salicylate hydroxylase encoded by *pahG’* in strain J3 and the salicylate hydroxylase encoded by *pahG* in strain E4. 1-hydroxy-2-naphthoic acid has been identified as the most important intermediate in the constructed degradation of phenanthrene, which may also limit the degradation rate of phenanthrene by PAH-degrading pure cultures [6,12].

In strain J3, 1,2-dihydroxynaphthalene was further converted into salicylic acid through the sequential actions of *pahC* and *pahDEF*. Similarly, strain J3 can metabolize salicylic acid into gentisic acid, followed by ring cleavage catalyzed by the functionally critical enzyme G12O, ultimately channeling the metabolites into the tricarboxylic acid (TCA) cycle. Additionally, J3 possesses a parallel phthalate pathway terminating with P34O-mediated cleavage. Crucially, the functional annotation reveals a distinct metabolic division of labor within the constructed consortium, characterized by a cross-feeding mechanism. Salicylic acid generated by strain J3 acts as another cross-feeding intermediate and is shuttled to its partner, strain E4. Upon integration, strain E4 effectively converts this intermediate into catechol via *pahG*. In strain E4, catechol can undergo ring cleavage catalyzed by C23O to form 2-hydroxymuconic semialdehyde. The C23O pathway has been reported to be the most important pathway in phenanthrene downstream degradation in PAH-degrading *Sphingomonas* [31,32], *Pseudomonas* [33], *Marinobacter* [34] and *Burkholderia* [35]. Hence, the presence of multiple downstream pathways is likely one of the key contributing factors to the high phenanthrene degradation efficiency and robust environmental adaptability of the constructed consortium NOMA.

### 3.6. Genome-Scale Metabolic Modeling and Potential Cross-Feeding Interactions in NOMA

The enhanced PAH degradation efficiency of bacterial consortia can arise from several interconnected mechanisms, including the mitigation of substrate toxicity, the activation of alternative catabolic pathways, and metabolic cross-feeding [36]. Because traditional empirical methods frequently fail to fully capture these complex intercellular dynamics [37], to further investigate the potential metabolic interactions between the two strains, genome-scale metabolic models (GSMMs) of *Novosphingobium* sp. J3 and *Martelella* sp. E4 were initially reconstructed using the CarveMe pipeline through the iNAP2 platform [38,39,40].

The analysis revealed an asymmetric and bidirectional pattern of metabolic complementarity between J3 and E4. The complementarity score corresponding to potential metabolic support from E4 to J3 was 0.1720, whereas that corresponding to the reciprocal J3 to E4 interaction was 0.1034. This difference indicates that the potential metabolic complementarity between the two strains is direction-dependent, with a stronger network-level contribution predicted from E4 to J3.

PhyloMInt-based analysis of the final curated models identified 42 nonredundant potentially transferable metabolites (PTMs), including 27 candidates in the E4 to J3 direction and 15 in the J3 to E4 direction. Among all PTMs, 13 were represented in the extracellular compartment (e0), 1 in the periplasmic compartment (p0), and 28 in the cytosolic compartment (c0). Because intracellular and periplasmic metabolites cannot be directly interpreted as extracellular exchanges, the complete compartment-resolved PTM list is provided in Table S5. Seven extracellular PTMs with clearer functional relevance to aromatic degradation, nutrient supply, or cofactor metabolism were selected for presentation in Table 2.

In the J3 to E4 direction, 1-hydroxy-2-naphthoate and salicylate were identified as key extracellular PTM candidates. Both compounds are consistent with the intermediates detected by GC–MS and with the proposed phenanthrene degradation pathway. Genome annotation further showed that strain E4 possesses salicylate 1-monooxygenase and catechol 2,3-dioxygenase, supporting its genetic potential for downstream utilization of salicylate-derived aromatic compounds. These results therefore support a model in which J3 performs upstream phenanthrene transformation and may provide aromatic intermediates that can subsequently enter the downstream catabolic network of E4.

In the reciprocal E4 to J3 direction, thiamine emerged as the most functionally relevant extracellular PTM, together with D-glucose, L-glutamate, Cys-Gly, and heme. Thiamine is particularly noteworthy because it functions as a precursor of thiamine pyrophosphate, an essential cofactor involved in central carbon metabolism and aerobic respiration. Previous studies have demonstrated that thiamine exchange can mediate cooperative interactions in pollutant-degrading microbial communities. Huang et al. [41] showed that thiamine produced by *Escherichia coli* K12 supported the growth and tetrahydrofuran degradation of the thiamine-auxotrophic degrader *Rhodococcus ruber* ZM07 through extracellular metabolite exchange, while Xu et al. [42] identified thiamine-mediated metabolic interactions as an important component of bacterial community assembly and environmental adaptation and further implicated thiamine exchange in a bromoxynil-degrading synthetic community.

Taken together, the GSMM and PhyloMInt analyses suggest a bidirectional but functionally asymmetric pattern of metabolic complementarity within consortium NOMA. J3 may primarily contribute PAH-derived aromatic intermediates, including 1-hydroxy-2-naphthoate and salicylate, that can potentially be utilized by E4, whereas E4 may provide complementary nutritional and cofactor-related support to J3, with thiamine representing a particularly relevant candidate. This reciprocal organization provides a plausible network-level explanation for the enhanced degradation performance of the consortium.

### 3.7. Annotation of Catabolic and Adaptive Genes in Consortium NOMA

**Figure 8:**
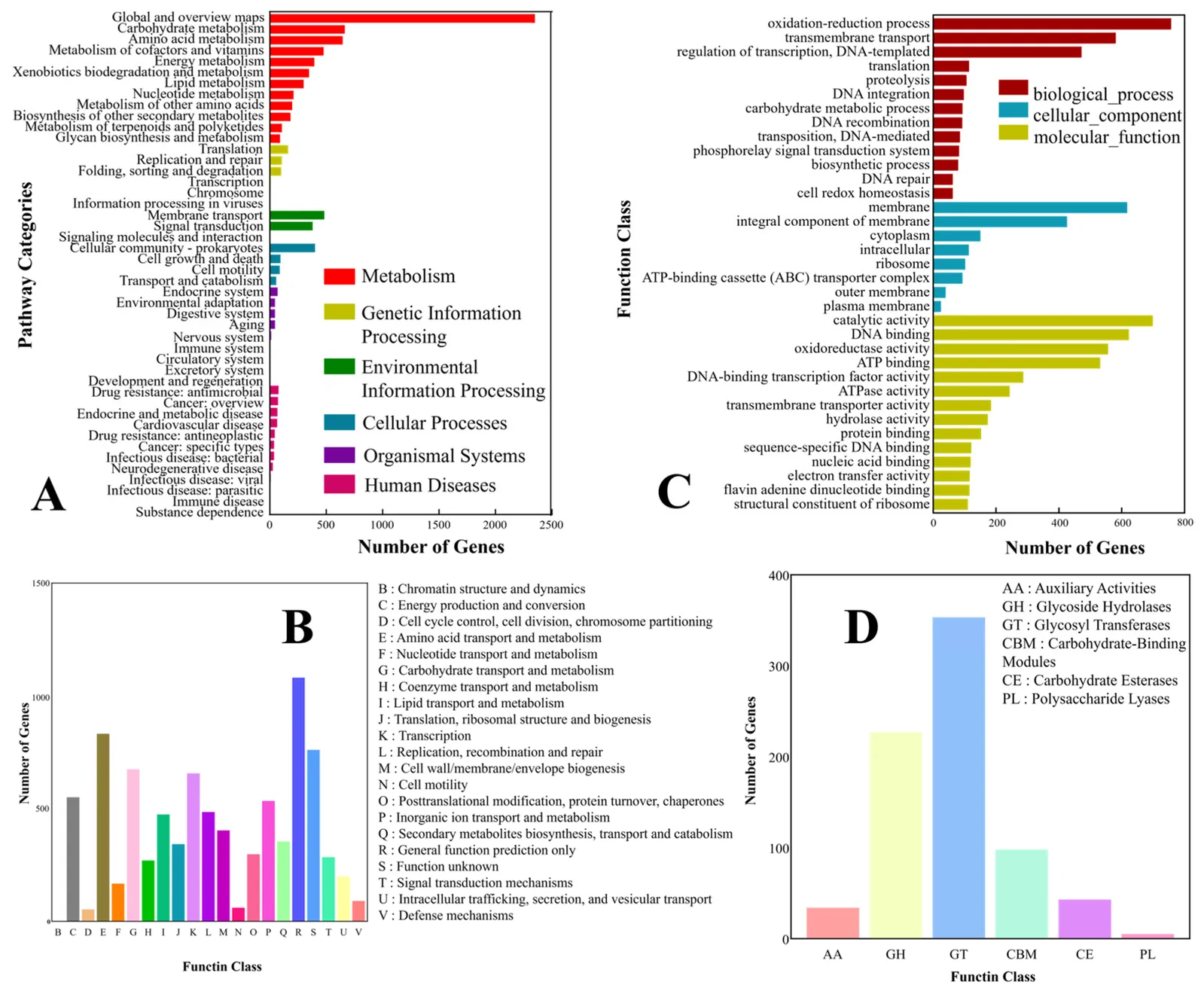
Functional annotation of genes in consortium NOMA based on (**A**) Kyoto Encyclopedia of Genes and Genomes (KEGG) pathways, (**B**) Clusters of Orthologous Groups (COG) functional categories, (**C**) Gene Ontology (GO) terms, and (**D**) carbohydrate-active enzymes annotated using the CAZy database.

Genomic classification via the KEGG database revealed a high representation of genes within the “Environmental Information Processing” category, specifically those governing “Membrane transport” and “Signal transduction” (Figure 8A). This targeted genomic allocation equips consortium NOMA to counter severe osmotic pressure variations and toxicological stress in saline habitats. In particular, efficient transport systems may facilitate the regulation of intracellular ion concentrations and the uptake or efflux of environmentally relevant compounds, whereas signal transduction pathways enable rapid physiological responses to changes in external conditions. Subsequent functional profiling using the COG database highlighted active core biochemical pathways, such as those involved in translation, replication, and carbohydrate metabolism, as well as abundant stress response mechanisms (Figure 8B). The coexistence of essential cellular functions and stress-related processes suggests that NOMA can maintain basic metabolic activity while dynamically reallocating cellular resources under adverse conditions. Complementing these findings, GO sequence annotations [43] confirmed a concentrated functional investment in catalytic activity and environmental perception (Figure 8C). Together, these features enable the microflora to process complex extracellular signals and maintain stable intracellular homeostasis under extreme environmental variations. Furthermore, protein sequence evaluation against the CAZy database demonstrated a genetic capacity for both complex glycan degradation and polysaccharide assembly (Figure 8D). These specific metabolic traits facilitate the exploitation of flexible carbon sources and drive multicellular defense behaviors, including biofilm formation, thereby potentially improving community persistence and functional stability in fluctuating saline environments.

Collectively, these genomic attributes reveal profound environmental plasticity and superior resource utilization efficiency, providing a potentially robust biological framework for phenanthrene degradation under variable physicochemical conditions.

## 4. Conclusions

In this study, a highly efficient halophilic constructed consortium, designated NOMA, was successfully constructed from *Novosphingobium* sp. J3 and *Martelella* sp. E4 based on the bottom-up method. Consortium NOMA was able to degrade 100 mg/L phenanthrene in 7 days at 5% salinity. It retained phenanthrene-degradation activity across the tested salinity range of 1–20%, although its performance declined markedly at the highest salinities of 15–20%. Based on GC–MS detection and genomic information analysis, a cross-feeding model of phenanthrene degradation between strain J3 and strain E4 was proposed, with multiple downstream pathways. 1-hydroxy-2-naphthoic acid and salicylic acid were identified as the key intermediates in this cross-feeding process. GSMM analysis suggested that strain J3 may provide aromatic intermediates to strain E4, whereas strain E4 may provide complementary extracellular metabolites, including thiamine, D-glucose, L-glutamate, Cys-Gly, and heme, to strain J3. Moreover, strain E4 may contribute to enhanced phenanthrene degradation by facilitating the downstream transformation of aromatic intermediates and potentially limiting their accumulation. To our knowledge, this is the first report on a proposed cross-feeding model of phenanthrene by halophiles; moreover, 1-hydroxy-2-naphthoic acid and salicylic acid were both identified as key metabolites in the cross-feeding process.

Nevertheless, the proposed cross-feeding model between strains J3 and E4 was mainly inferred from genomic annotation, metabolite detection, and topology-based metabolic modeling, and direct interspecies metabolite transfer remains to be experimentally verified. In addition, the present experiments were conducted in a defined liquid medium, and Cd^2+^ removal was not measured. Future studies should further verify the proposed metabolic interactions using approaches such as isotope tracing or strain-resolved metabolite analysis, and evaluate the stability and degradation performance of consortium NOMA in environmentally relevant matrices, including saline sediments, soils, and wastewater, under more complex contaminant conditions.

## Figures and Tables

**Table 1:** The utilization ability of intermediates by strain E4 and J3.

Carbon Source	PHE	1H2N	Salicylic Acid	Gentisate	Catechol	Protocatechuic Acid
Strain J3	√	√	√	√	-	√
Strain E4	-	-	√	-	√	-

PHE stands for phenanthrene, 1H2N stands for 1-hydroxy-2-naphthoic acid. “√” indicates that the strain can utilize the compound as a carbon source, whereas “-” indicates that it cannot.

**Table 2:** Key extracellularly represented potentially transferable metabolites predicted in the NOMA system.

Donor	Receptor	Metabolite ID	Metabolite
J3	E4	cpd02045	1-Hydroxy-2-naphthoate
J3	E4	cpd00599	salicylate
E4	J3	cpd00305	Thiamine
E4	J3	cpd00027	D-Glucose
E4	J3	cpd00023	L-Glutamate
E4	J3	cpd01017	Cys-Gly
E4	J3	cpd00028	Heme

## Data Availability

The original contributions presented in this study are included in the article and Supplementary Materials. Further inquiries can be directed to the corresponding author.

## Supplementary Materials

The following supporting information can be downloaded at: https://www.mdpi.com/article/10.3390/microorganisms14081833/s1. Table S1: Annotation of PAH-degrading genes in consortium NOMA; Table S2: Annotation of genes in gene cluster in *Novosphingobium* sp. J3; Table S3: Annotation of heavy metal resistance genes in consortium NOMA; Table S4: Metabolite detection using GC-MS in phenanthrene degrading process; Table S5: Nonredundant potentially transferable metabolites predicted by PhyloMInt in NOMA system grouped by model compartment; Table S6: Apparent kinetic parameters for phenanthrene degradation by strain J3 and consortium NOMA under different experimental conditions.
