# Supplementary material for: A Proposed Cross-Feeding Model of Phenanthrene Degradation by Constructed Halophilic Consortium NOMA
Source: Microorganisms. 2026 Aug 19;14(8):1833. doi: 10.3390/microorganisms14081833 (PMC13515625; doi:10.3390/microorganisms14081833)
Supplement: Supplementary file 1 [file microorganisms-14-01833-s001.zip › microorganisms-4477220-supplementary.pdf]

Tables

Table S1 Annotation of PAH-degrading genes in consortium NOMA.

Table S2 Annotation of genes in gene cluster in *Novosphingobium* sp. J3.

Table S3 Annotation of heavy metal resistance genes in consortium NOMA.

Table S4 Metabolite detection using GC-MS in phenanthrene degrading process.

Table S5 Nonredundant potentially transferable metabolites predicted by PhyloMInt in NOMA system grouped by model compartment.

Table S6 Apparent kinetic parameters for phenanthrene degradation by strain J3 and consortium NOMA under different experimental conditions.

Table S1 Annotation of PAH-degrading genes in consortium NOMA.

Strain	Gene	Length	Encoding enzyme	Bacteria with most similarity/E-value in Conserved Domain Database	Accession
J3	*pahAc*	450	Aromatic ring-hydroxylating dioxygenase subunit alpha	*Pelagerythrobacter aerophilus*(100%)	WP_449014960.1
	pahB	267	2,3-dihydroxy-2,3-dihydrophen-ylpropionate dehydrogenase	*Sphingobium* sp. C100 (98.84%)	ETI62774.1
	pahC	301	1,2-dihydroxynaphthalene dioxygenase	*Pseudomonas veronii*（62.16%）	WP_141123765.1
	pahD	198	2-hydroxychromene-2-carboxylate isomerase	*Novosphingobium mangrovisedimenti* (100%)	WP_260047870.1
	pahE	329	trans-o-hydroxybenzylidenepyruvate hydratase	*Sphingobium* (82.93%)	WP_026149928.1
	pahF	505	salicylaldehyde dehydrogenase	*Novosphingobium indicum* (96.47%)	GGN61216.1
	pahG	418	salicylate 5-hydroxylase	*Sphingopyxis* sp. BSNA05 (77.81%)	NRD91094.1
	pahX	350	gentisate 1,2-dioxygenase	*Pelagerythrobacter aerophilus* (100%)	WP_449014971.1
	pcaG	272	protocatechuate 3,4-dioxygenase	*Pseudomonadota bacterium* (63.81%)	MES2337698.1
	phdJ	327	trans-2'-carboxybenzalpyruvate hydratase-aldolase	3.06×10^{-177}	cd00952
E4	*pahG*	441	salicylate hydroxylase	*Rhizobium* sp. (54.78%)	HCL65690.1
	nahH	308	catechol 2,3-dioxygenase	*Novosphingobium mangrovisedimenti* (100%)	WP_260047927.1

Table S2 Annotation of genes in gene cluster in *Novosphingobium* sp. J3.

Name	Size	Encoding enzyme	Bacteria with most similarity (identity)	Accession
pahC	301	1,2-dihydroxynaphthalene dioxygenase	*Pseudomonas veronii*（62.16%）	WP_141123765
ORF1	824	TonB-dependent receptor	*Sphingobium algorifonticola*(82.29%)	WP_241242031
pahD	199	2-hydroxychromene-2-carboxylate isomerase	*Pelagerythrobacter aerophilus*(100%)	WP_449014965
pahE	327	trans-o-hydroxybenzylidenepyruvate hydratase-aldolase	*Solimonas aquatica*(72.31%)	SEP74235
pahAc	458	Aromatic ring-hydroxylating dioxygenase subunit alpha	*Pelagerythrobacter aerophilus*(100%)	WP_449014966
pahAd	203	Aromatic ring-hydroxylating dioxygenase subunit beta	*Pelagerythrobacter aerophilus*(100%)	WP_449014967
pahB	292	2,3-dihydroxy-2,3-dihydrophenylpropionate dehydrogenase	*Massilia* sp.(63.24%)	MDB5916472
ORF2	305	fumarylacetoacetate hydrolase family protein	*Pelagerythrobacter aerophilus*(99.66%)	WP_449014969
pahAa	346	naphthalene dioxygenase ferredoxin reductase	*Pelagerythrobacter aerophilus*(100%)	WP_449014970
pahX	372	gentisate 1,2-dioxygenase	*Pelagerythrobacter aerophilus*(100%)	WP_449014971
ORF3	279	fumarylacetoacetate hydrolase family protein	*Pelagerythrobacter aerophilus*(100%)	WP_449014972

Table S3 Annotation of heavy metal resistance genes in consortium NOMA.

**Gene Name**	**Length**	**Putative Function**	**Similarity**	**Accession**
cadA	707	cadmium-translocating P-type ATPase	*Microvirga* sp.(93.06%)	MCG6122998.1
czcA	1045	CusA/CzcA family heavy metal efflux RND transporter	*Pseudomonadota bacterium*(85.80%)	MED5545921.1
zntA	728	Cd^{2+}/Zn^{2+}-exporting ATPase	*Aquamicrobium terrae* (68.98%)	CAM5330042.1
copA	762	Copper-transporting P-type ATPase	*Hartmannibacter diazotrophicus* (83.84%)	SON55382.1
chrA	443	chromate efflux transporter	*Martelella* sp. AMO21009(95.59%)	WP_448464453.1
merA	476	mercury(II) reductase	*Aquamicrobium defluvii*(99.78%)	WP_425359071.1
merT	183	mercuric transporter MerT family protein	*Aquamicrobium defluvii*(99.19%)	WP_425359070.1
arsC	144	arsenate reductase	*Martelella* sp. FLE1502 (97.08%)	WP_448474993.1
acr3	357	ACR3 family arsenite efflux transporter	*Martelella* sp. FLE1502 (99.16%)	WP_448472347.1
smtB	100	ArsR/SmtB family transcription factor	*Pseudochelatococcus* sp. B33(75.00%)	WP_449311412.1

Table S4 Metabolite detection using GC-MS in phenanthrene degrading process.

No.	Detection time (min)	Major ions (M^+)	Compounds (TMS-)
1	36’22”	178 (M^+), 152, 88, 76, 62	Phenanthrene
2	35’16”	332 (M^+), 317, 243, 185, 170, 147, 73	1-hydroxy-2-naphthoic acid
3	34’31”	304 (M^+)，259，247，217，201，190，147，117，73	1,2-dihydroxynaphthalene
4	24’37”	310 (M^+), 295, 221, 163, 147, 73	Phthalic acid
5	20’58”	267 (M^+), 209, 193 , 149, 135 , 91, 73	Salicylic acid
6	23’51”	254 (M^+), 239, 209, 147, 91, 73	Catechol

Table S5 Nonredundant potentially transferable metabolites predicted by PhyloMInt in NOMA system grouped by model compartment.

Donor	Receptor	Metabolite ID	Metabolite	Compartment
J3	E4	cpd00149	Co^{2+}	e0
J3	E4	cpd00599	salicylate	e0
J3	E4	cpd02045	1-Hydroxy-2-naphthoate	e0
J3	E4	R__45__3hpbcoa__45__c	3 Hydroxyphenylbutanoyl CoA	c0
J3	E4	R__45__3hpdecacoa__45__c	3 Hydroxyphenyldecanoyl CoA	c0
J3	E4	R__45__3hphpcoa__45__c	3 Hydroxyphenylheptanoyl CoA	c0
J3	E4	R__45__3hpnonacoa__45__c	3 Hydroxyphenylnonanoyl CoA	c0
J3	E4	R__45__3hpoctacoa__45__c	3 Hydroxyphenyloctanoyl CoA	c0
J3	E4	R__45__3hptcoa__45__c	3 Hydroxpypentoyl CoA	c0
J3	E4	cpd00227	L-Homoserine	c0
J3	E4	cpd00616	Pretyrosine	c0
J3	E4	cpd00712	2-Dehydropantoate	c0
J3	E4	cpd02016	N-Ribosylnicotinamide	c0
J3	E4	cpd06708	Benzylsuccinate	c0
J3	E4	cpd06711	(Hydroxymethylphenyl)succinyl-CoA	c0
E4	J3	cpd00023	L-Glutamate	e0
E4	J3	cpd00027	D-Glucose	e0
E4	J3	cpd00028	Heme	e0
E4	J3	cpd00039	L-Lysine	e0
E4	J3	cpd00081	Sulfite	e0
E4	J3	cpd00205	K^{+}	e0
E4	J3	cpd00254	Mg^{2+}	e0
E4	J3	cpd00268	Thiosulfate	e0
E4	J3	cpd00305	Thiamine	e0
E4	J3	cpd01017	Cys-Gly	e0
E4	J3	cpd00041	L-Aspartate	p0
E4	J3	2ahethmpp__45__c	2-(alpha-Hydroxyethyl)thiamine diphosphate	c0
E4	J3	cpd00064	Ornithine	c0
E4	J3	cpd00186	D-Glutamate	c0
E4	J3	cpd00224	L-Arabinose	c0
E4	J3	cpd00282	S-Dihydroorotate	c0
E4	J3	cpd00322	L-Isoleucine	c0
E4	J3	cpd00334	L-Lactaldehyde	c0
E4	J3	cpd00434	4-Aminobutanal	c0
E4	J3	cpd00486	Indoleacetaldehyde	c0
E4	J3	cpd00802	D-fructose-1-phosphate	c0
E4	J3	cpd00822	O-Succinyl-L-homoserine	c0
E4	J3	cpd01034	Toluene	c0
E4	J3	cpd02095	L-2,4-Diaminobutyrate	c0

E4	J3	cpd15419	CDP-1,2-dihexadecanoylglycerol	c0
E4	J3	cpd15494	Maltoheptaose	c0
E4	J3	glc__45__aD__45__c	α-D-Glucose	c0

Table S6 Apparent kinetic parameters for phenanthrene degradation by strain J3 and consortium NOMA under different experimental conditions.

Experimental group	Condition	Selected kinetic order	Rate constant (k)	Unit	R^2	Half-life (d)	Notes
Culture comparison	J3	Zero-order	9.450	mg L^{-1} d^{-1}	0.862	5.29	Monoculture
Culture comparison	NOMA	Zero-order	15.418	mg L^{-1} d^{-1}	0.884	3.24	Co-culture
Salinity	1%	Zero-order	14.480	mg L^{-1} d^{-1}	0.959	3.45	NOMA
Salinity	3%	Zero-order	14.265	mg L^{-1} d^{-1}	0.897	3.51	NOMA
Salinity	5%	Zero-order	14.508	mg L^{-1} d^{-1}	0.897	3.45	NOMA
Salinity	10%	Zero-order	11.330	mg L^{-1} d^{-1}	0.835	4.41	NOMA
Salinity	15%	Zero-order	5.681	mg L^{-1} d^{-1}	0.806	8.80	NOMA
Salinity	20%	Zero-order	2.333	mg L^{-1} d^{-1}	0.832	21.43	NOMA
Cd^{2+}	0 mg/L	Zero-order	13.900	mg L^{-1} d^{-1}	0.816	3.60	NOMA
Cd^{2+}	0.5 mg/L	Zero-order	15.080	mg L^{-1} d^{-1}	0.866	3.32	NOMA
Cd^{2+}	10 mg/L	Zero-order	12.530	mg L^{-1} d^{-1}	0.812	3.99	NOMA
Cd^{2+}	25 mg/L	Zero-order	9.488	mg L^{-1} d^{-1}	0.794	5.27	NOMA
Cd^{2+}	50 mg/L	Zero-order	8.007	mg L^{-1} d^{-1}	0.769	6.24	NOMA
